# Integrative Analysis Constructs an Extracellular Matrix-Associated Gene Signature for the Prediction of Survival and Tumor Immunity in Lung Adenocarcinoma

**DOI:** 10.3389/fcell.2022.835043

**Published:** 2022-04-26

**Authors:** Lingyan Xiao, Qian Li, Yongbiao Huang, Zhijie Fan, Wan Qin, Bo Liu, Xianglin Yuan

**Affiliations:** ^1^ Department of Oncology, Tongji Hospital, Tongji Medical College, Huazhong University of Science and Technology, Wuhan, China; ^2^ Department of Pathophysiology, School of Basic Medicine, Tongji Medical College, Huazhong University of Science and Technology, Wuhan, China

**Keywords:** lung adenocarcinoma, extracellar matrix, prognostic signature, tumor micoenvironment, immunotharapy

## Abstract

**Background:** Lung adenocarcinoma (LUAD) accounts for the majority of lung cancers, and the survival of patients with advanced LUAD is poor. The extracellular matrix (ECM) is a fundamental component of the tumor microenvironment (TME) that determines the oncogenesis and antitumor immunity of solid tumors. However, the prognostic value of extracellular matrix-related genes (ERGs) in LUAD remains unexplored. Therefore, this study is aimed to explore the prognostic value of ERGs in LUAD and establish a classification system to predict the survival of patients with LUAD.

**Methods:** LUAD samples from The Cancer Genome Atlas (TCGA) and GSE37745 were used as discovery and validation cohorts, respectively. Prognostic ERGs were identified by univariate Cox analysis and used to construct a prognostic signature by Least Absolute Shrinkage and Selection Operator (LASSO) regression analysis. The extracellular matrix-related score (ECMRS) of each patient was calculated according to the prognostic signature and used to classify patients into high- and low-risk groups. The prognostic performance of the signature was evaluated using Kaplan–Meier curves, Cox regression analyses, and ROC curves. The relationship between ECMRS and tumor immunity was determined using stepwise analyses. A nomogram based on the signature was established for the convenience of use in the clinical practice. The prognostic genes were validated in multiple databases and clinical specimens by qRT-PCR.

**Results:** A prognostic signature based on eight ERGs (*FERMT1*, *CTSV*, *CPS1*, *ENTPD2*, *SERPINB5*, *ITGA8*, *ADAMTS8*, and *LYPD3*) was constructed. Patients with higher ECMRS had poorer survival, lower immune scores, and higher tumor purity in both the discovery and validation cohorts. The predictive power of the signature was independent of the clinicopathological parameters, and the nomogram could also predict survival precisely.

**Conclusions:** We constructed an ECM-related gene signature which can be used to predict survival and tumor immunity in patients with LUAD. This signature can serve as a novel prognostic indicator and therapeutic target in LUAD.

## Introduction

With approximately 1.8 million new cases diagnosed annually, lung cancer remains the primary cause of cancer-related death globally (Ferlay et al., 2015). Non-small-cell lung carcinoma is the main histological type of lung cancer, and LUAD is the most common subtype. The reported 5-years survival rate of non-small-cell lung cancer patients across all stages of the disease is 26% (American Cancer Society, 2017). The rising incidence of lung cancer and poor survival of patients call for robust biomarkers to predict patient outcomes.

The extracellular matrix (ECM) is defined as the acellular component of tissues that can provide biochemical and biophysical support for cells. ECM genes can be broadly divided into core- and matrisome-related molecules. The major components of the ECM include collagens, glycoproteins, proteoglycans, and other molecules, such as hyaluronan and galectin. As a fundamental component of organisms, the ECM is essential for organ development and cell communication. The ECM is also an important constituent of solid tumors and can be altered through time and space to create a microenvironment that facilitates oncogenesis and progression (Peng et al., 2017; Pearce et al., 2018; Mierke, 2019; Cox, 2021). Alterations in the components or organization of the ECM can modulate a series of signaling pathways that regulate cell proliferation, differentiation, migration, and other behaviors (Nebuloni et al., 2016; Peng et al., 2017; Fattet et al., 2020). Some studies have revealed the prognostic role of ECM-related genes in cancer. An ECM-associated gene signature has been found to correlate with patient outcomes in early-stage non-small-cell lung cancer (Lim et al., 2017). Downregulation of lumican and decorin has been shown to be related to poor prognosis in breast malignancies (Troup et al., 2003).

Immunotherapy is an emerging and effective therapy for lung cancer, but the main challenge in immunotherapy is the low response rate of patients. Recent years have seen explosive growth in studies exploring approaches to predicting and augmenting the response to immunotherapy. Pancancer analysis has revealed that transforming growth factor (TGF)-β-associated ECM genes are reliable predictors of immunotherapy response (Chakravarthy et al., 2018).

Although ECM is significant in tumorigenesis and can be a potent indicator of survival, no attempt has been made to comprehensively explore the prognostic role of ECM-associated genes in LUAD. Therefore, this study aimed to explore the prognostic value of ERGs in LUAD and develop a classification system, based on the expression level of ERGs, to predict the survival of patients with LUAD.

## Materials and Methods

### Data Acquisition and Differential Analysis

Transcriptional data were obtained from TCGA-LUAD dataset, GSE37745, GSE32863, and GSE43458. ERGs were identified from the Gene Ontology website (http://geneontology.org/) with the key word “extracellular matrix.” The list of ERGs is provided in Supplementary Table 1. The package “edgeR” was used to preprocess the expression data in TCGA (Robinson et al., 2010; McCarthy et al., 2012; Chen et al., 2016), including discarding genes with expression less than five in all samples and normalizing the expression data. Genes that met the filtering criteria of adjusted *p* value (false discovery rate) < 0.05 and |log_2_ fold change| >2.0 were considered dysregulated ERGs.

### Identification of Prognostic Extracellular Matrix-Related Genes and Construction of a Prognostic Signature

To screen prognostic genes from the differentially expressed ERGs, univariate Cox regression analysis was conducted with the “survival” package (Therneau et al., 2000). As a common machine learning method, LASSO Cox regression analysis can properly handle multicollinearity and is frequently applied to construct prognostic signatures (Gui and Li, 2005; Wang and Liu, 2020). Thus, LASSO regression analysis was performed with the “glmnet” package to screen prognostic genes further and create a prognostic signature that was presented as a formula (Friedman et al., 2010). The ECMRS of each sample was calculated using regression coefficients and mRNA levels of prognostic ERGs in the formula. The classification of patients into high-risk and low-risk groups was based on the median ECMRS.

### Validation of the Prognostic Signature

The discovery cohort was randomly divided into two subsets (N1 = 240, N2 = 239) to test the prognostic potential of the signature. The prognostic performance of the signature was further evaluated in the entire discovery cohort (N = 479) and external testing cohort (GSE37745, N = 196). Kaplan–Meier survival curves were generated to compare overall survival (OS) between the high- and low-risk patients based on the log-rank test. Next, univariate and multivariate Cox regression analyses were conducted to assess the effects of ECMRS on OS by using the “survival” packages (Therneau et al., 2000). ROC curves were created to evaluate the power of the signature in OS prediction with the “timeROC” package (Blanche et al., 2013).

### Evaluation of Association Between ECMRS and Clinicopathological Variables

The difference in ECMRS among patients stratified by clinical parameters was evaluated to elucidate the effect of ECMRS on cancer progression. Moreover, the survival probability of LUAD patients stratified by clinicopathological variables was assessed using Kaplan–Meier curves to explore whether the prognostic value of our signature changed with clinical parameters.

### Assessment of the Relationship Between ECMRS and Immunophenotypes

ESTIMATE is an algorithmic tool that can calculate tumor purity and the abundance of cells in the TME, including immune cells and stromal cells (Yoshihara et al., 2013). Here, the ESTIMATE algorithm was run to obtain the immune score, stromal score, and ESTIMATE score of each patient with the “estimate” package. Immune cells infiltrating the TME of TCGA-LUAD were identified from TIMER (Li et al., 2016; Li et al., 2020a) (http://timer.comp-genomics.org/). ssGSEA was performed using the R package “GSVA” to further identify the related immune processes of the signature in both TCGA and GEO cohorts (Subramanian et al., 2005; Hänzelmann et al., 2013). The relationship between the prognostic signature and immune checkpoint gene expression was also explored. Comparison of all these results between the low- and high-risk patients was done using Wilcox test.

### Visualization of the Prognostic Signature

To visualize our prognostic signature, a nomogram was established based on ECMRS for survival prediction by using the “rms package.” Additionally, calibration curves at 3 and 5 years were created to show the predictive accuracy of the nomogram.

### Validation of the Prognostic Extracellular Matrix-Related Genes

Differential expression analysis was performed in GSE43458 [N (Normal) = 30, N (Tumor) = 80] and GSE32863 [N (Normal) = 58, N (Tumor) = 58] with the “limma” package to verify whether the ERGs in the signature were also differentially expressed in other datasets (Ritchie et al., 2015; Phipson et al., 2016). The Human Protein Atlas (HPA) database is a tool developed to map the proteome of human tissues and cancers (https://www.proteinatlas.org/) (Uhlén et al., 2015; Uhlen et al., 2017). Immunohistochemistry images of LUAD and normal lung tissues were acquired from the HPA database to validate the protein expression of the prognostic ERGs. The effects of these ERGs on the survival of patients with LUAD were validated using Kaplan–Meier plotter (http://kmplot.com/analysis/) (Győrffy, 2021).

### Clinical Specimen Collection

Human lung adenocarcinoma tissues and paired peritumoral lung tissues were collected from 12 patients who underwent surgical resection at the Thoracic Surgery Department of Wuhan Tongji Hospital between March 2021 and August 2021. Written informed consent was obtained from all the 12 patients. All patients were histopathologically diagnosed with LUAD and had not received any antitumor therapy before surgery.

### RNA Extraction and qRT–PCR

Fresh LUAD tissues and peritumoral lung tissues were frozen in liquid nitrogen and stored at -80°C until RNA extraction. After thawing the tissues, total RNA was extracted using TRIzol reagent (Takara, Otsu, Japan) according to the manufacturer’s protocol. cDNA was synthesized using Hiscript@ Q RT SuperMix (Vazyme, Nanjing, China) and used for quantitative polymerase chain reaction detection with SYBR Green SuperMix (Vazyme, Nanjing, China). Quantitative reverse transcription polymerase chain reaction was performed under the following conditions: 95°C for 60 s, 40 cycles of 95°C for 5 s, and 50°C–60°C for 15 s mRNA levels were normalized to the expression of endogenous GAPDH. The primer sequences are provided in Supplementary Table 2.

## Results

### Dysregulated Extracellular Matrix-Related Genes of Lung Adenocarcinoma

**FIGURE 1:**
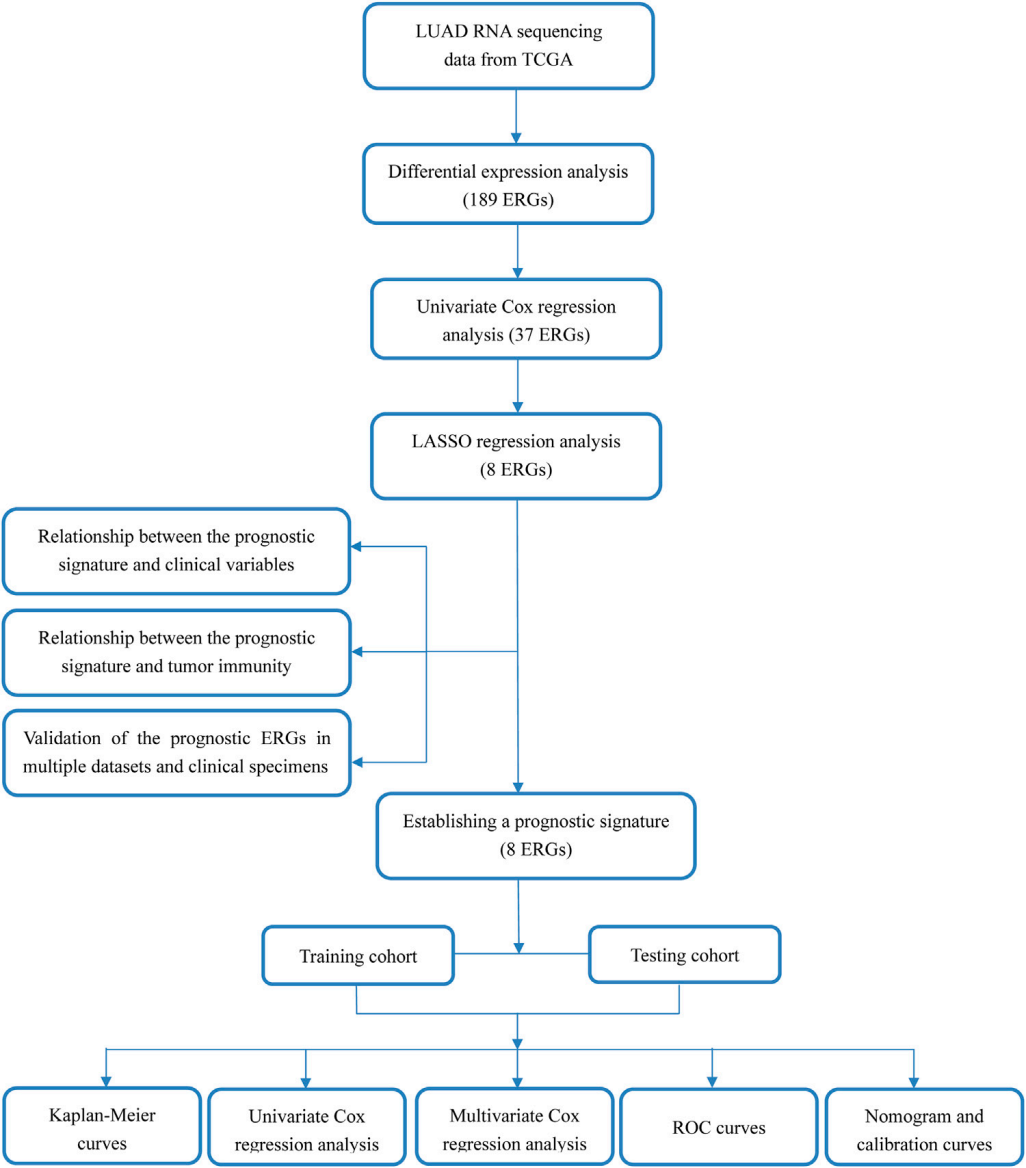
Work flow of this study.

**FIGURE 2:**
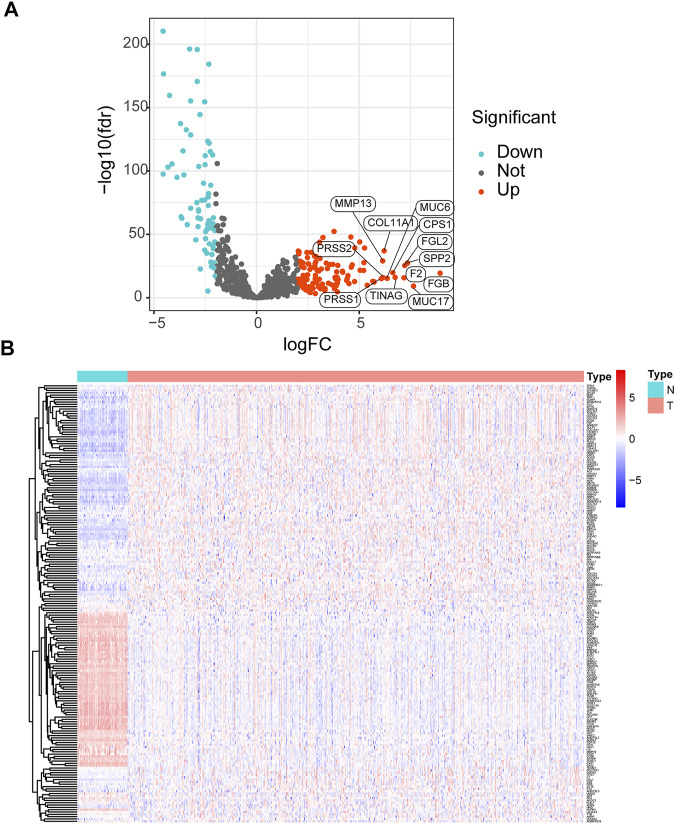
Analysis of differentially expressed ERGs **(A)** Volcano plot of differentially expressed ERGs, showing the FDR and log2-fold change of each gene **(B)** The heatmap shows the distribution of 189 differentially expressed ERGs between LUAD and normal lung tissues. ERGs = extracellular matrix-related genes; FDR = false discovery rate; LUAD = lung adenocarcinoma.

The workflow of the study is displayed in Figure 1. The expression data of 539 LUAD tissues and 59 normal lung tissues were downloaded and analyzed. A total of 189 dysregulated ERGs were identified in LUAD from 953 ERGs, with 119 upregulated genes and 70 downregulated genes (Figures 2A, B).

### Construction of a Prognostic Signature

The differentially expressed ERGs were subjected to univariate Cox regression analysis to obtain 37 prognostic genes (Table 1). The prognostic ERGs were further screened by LASSO regression analysis, and a prognostic signature was constructed based on these prognostic ERGs. The final signature was fit with eight key prognostic genes (*FERMT1*, *CTSV*, *CPS1*, *ENTPD2*, *SERPINB5*, *ITGA8*, *ADAMTS8*, and *LYPD3*). Among these prognostic genes, *FERMT1* (hazard ratio (HR) = 1.041, *p* < 0.001), *CTSV* (HR = 1.024, *p* = 0.010), *CPS1* (HR = 1.003, *p* = 0.001), *ENTPD2* (HR = 1.100, *p* < 0.001), *SERPINB5* (HR = 1.024, *p* < 0.001), and *LYPD3* (HR = 1.013, *p* < 0.001) were indicators of poor prognosis, whereas survival was positively affected by ITGA8 (HR = 0.860, *p* = 0.003) and *ADAMTS8* (HR = 0.771, *p* = 0.002) (Table 1). The predictive signature was created as a formula, and the ECMRS of each patient was estimated with regression co-efficient and mRNA levels of the prognostic ERGs in the formula (Supplementary Table 3). The median ECMRS was set as the threshold to classify patients into high and low-risk groups (Supplementary Table 4-5).

**TABLE 1:** Results of univariate cox regression analysis in the TCGA cohort.

Gene	HR	95% CI	Pvalue
ADAM12	1.054	1.009–1.101	0.019
ADAM8	1.012	1.000–1.024	0.050
ADAMTS8	0.771	0.655–0.908	0.002
AHSG	1.728	1.298–2.299	<0.001
APOC3	1.046	1.009–1.085	0.015
BCAN	1.049	1.000–1.101	0.050
CAV1	1.003	1.000–1.005	0.021
CAV2	1.016	1.005–1.026	0.003
CDK1	1.024	1.008–1.041	0.004
CLEC14A	0.973	0.948–0.998	0.036
COCH	1.043	1.004–1.083	0.029
COL22A1	1.106	1.025–1.194	0.010
COL6A6	0.751	0.609–0.926	0.008
COL7A1	1.027	1.001–1.054	0.043
COL9A1	1.916	1.184–3.101	0.008
CPS1	1.003	1.001–1.005	0.001
CTSV	1.024	1.006–1.043	0.010
ENTPD2	1.100	1.043–1.161	<0.001
F12	1.109	1.030–1.194	0.006
FAM107A	0.935	0.878–0.996	0.037
FBN2	1.037	1.018–1.055	<0.001
FERMT1	1.041	1.020–1.062	<0.001
FGA	1.000	1.000–1.001	0.039
FGF2	1.227	1.062–1.417	0.005
FOXF1	0.890	0.800–0.990	0.032
GDF10	0.887	0.794–0.990	0.033
HPSE2	0.416	0.206–0.840	0.014
ITGA8	0.860	0.780–0.949	0.003
JAM2	0.829	0.716–0.960	0.012
LYPD3	1.013	1.006–1.020	<0.001
MFAP4	0.993	0.988–0.998	0.006
RGCC	0.992	0.986–0.999	0.024
SERPINB5	1.024	1.011–1.036	<0.001
SMOC1	1.016	1.006–1.027	0.002
TEK	0.893	0.808–0.988	0.028
TINAG	1.144	1.052–1.245	0.002
ZG16	1.174	1.016–1.355	0.029

TCGA, the cancer genome atlas; HR, hazard ratio; CI, confidence interval.

### Validation of the Prognostic Signature in The Cancer Genome Atlas and Gene Expression Omnibus

**FIGURE 3:**
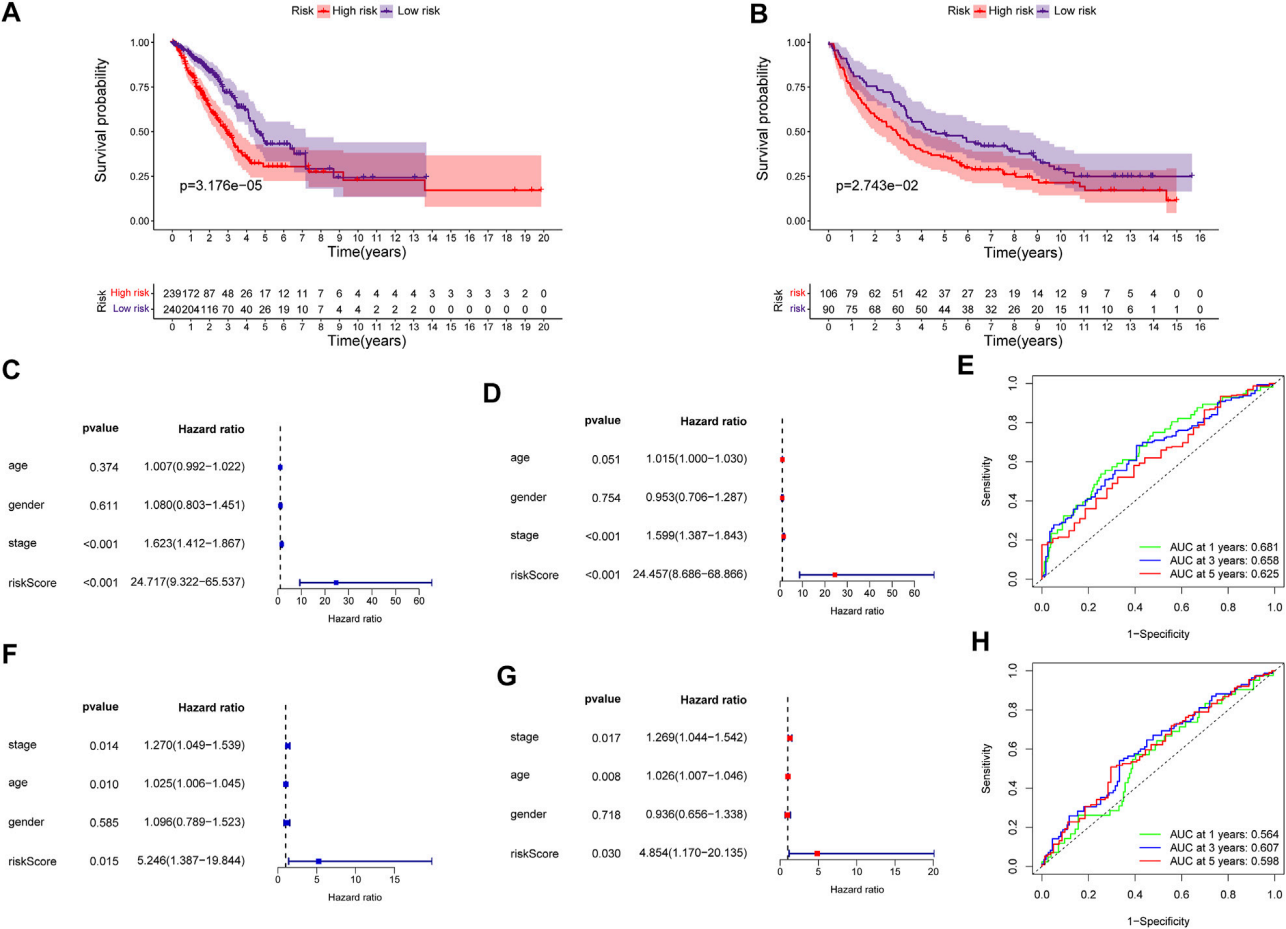
Validation of the prognostic signature in the entire TCGA and GEO cohorts **(A)** Kaplan–Meier survival curve of the high- and low-risk groups in the entire TCGA cohort **(B)** Kaplan–Meier survival curve of the high- and low-risk groups in the GEO cohort **(C)** Univariate Cox regression analysis of clinicopathological variables and ECMRS in the entire TCGA cohort **(D)** Multivariate Cox regression analysis of clinicopathological variables and ECMRS in the entire TCGA cohort **(E)** ROC curves of one‐, three‐ and five‐ years in the entire TCGA cohort indicating the predictive ability of ECMRS **(F)** Univariate Cox regression analysis of ECMRS and clinicopathological variables in the GEO cohort **(G)** Multivariate Cox regression analysis of ECMRS and clinicopathological variables in the GEO cohort **(H)** ROC curves of one‐, three‐ and five‐ years in the GEO cohort indicating the predictive ability of ECMRS. TCGA = The Cancer Genome Atlas; GEO = Gene Expression Omnibus; ECMRS = extracellular matrix-related score.

**FIGURE 4:**
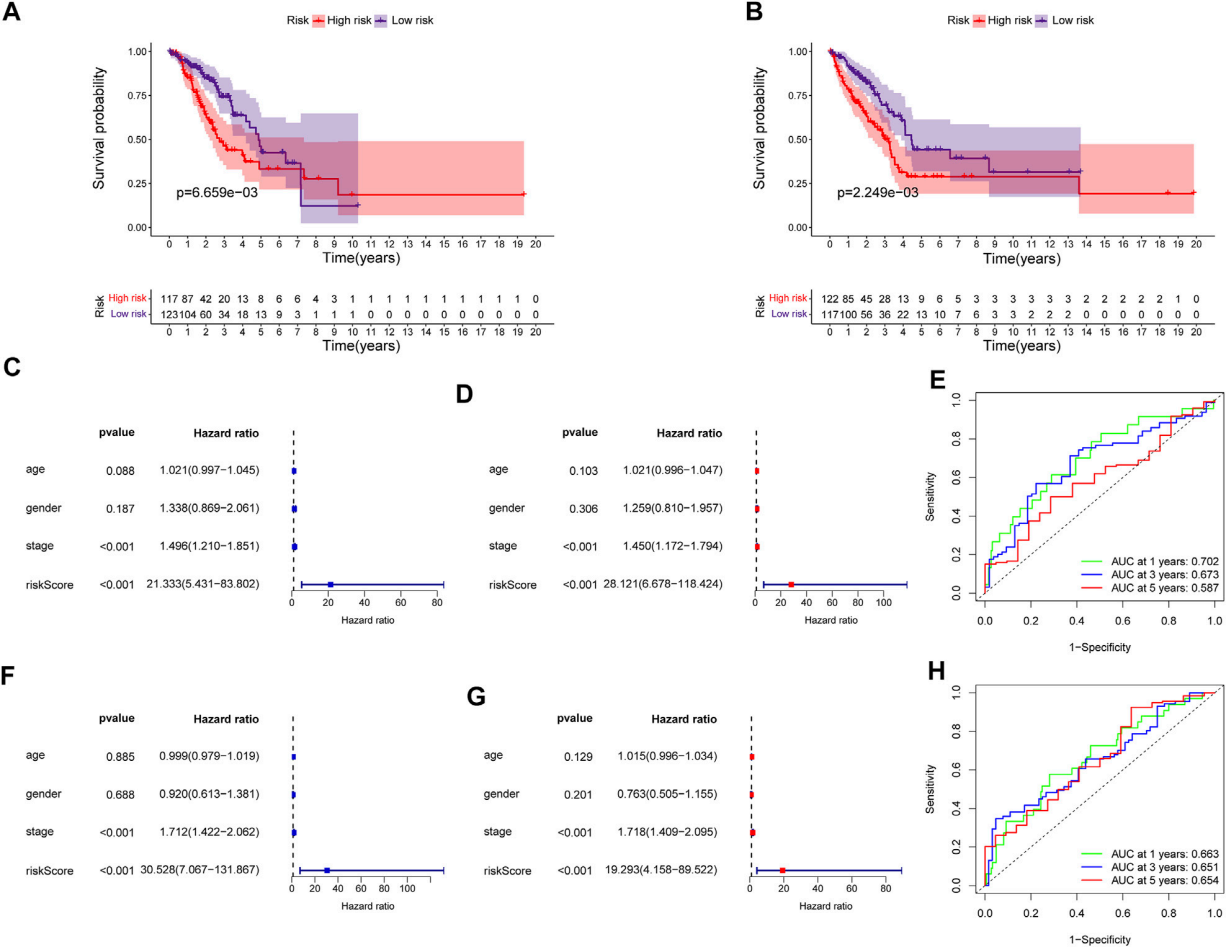
Validation of the prognostic signature in the two subsets of the TCGA cohort **(A)** Kaplan–Meier survival curve of the high- and low-risk groups in subset one of the TCGA cohort **(B)** Kaplan–Meier survival curve of the high- and low-risk groups in subset two of the TCGA cohort **(C)** Univariate Cox regression analysis of clinicopathological variables and ECMRS in subset one of the TCGA cohort **(D)** Multivariate Cox regression analysis of clinicopathological variables and ECMRS in subset one of the TCGA cohort **(E)** ROC curves of one‐, three‐ and five‐ years in subset one of the TCGA cohort indicating the predictive ability of ECMRS **(F)** Univariate Cox regression analysis of ECMRS and clinicopathological variables in subset two of the TCGA cohort **(G)** Multivariate Cox regression analysis of ECMRS and clinicopathological variables in subset two of the TCGA cohort **(H)** ROC curves of one‐, three‐ and five‐ years in subset two of the TCGA cohort indicating the predictive ability of ECMRS. TCGA = The Cancer Genome Atlas; GEO = Gene Expression Omnibus; ECMRS = extracellular matrix-related score.

The predictive power of the ECMRS was verified in the discovery cohort and GSE37745. Table 2 presents the clinical characteristics of the discovery cohort and GSE37745. Patients with low ECMRS were likely to live longer in both the discovery cohort (Figure 3A) and GSE37745 (Figure 3B). The results of univariate Cox regression analysis demonstrated that survival was adversely affected by ECMRS in the TCGA cohort (HR = 24.717, *p* < 0.001) (Figure 3C) and GSE37745 (HR = 5.246, *p* = 0.015) (Figure 3F). Consistent with the univariate Cox regression analysis, the results of multivariate Cox regression analysis also suggested that the adverse impact of ECMRS on prognosis in the discovery cohort (HR = 24.457, *p* value <0.001) (Figure 3D) and GSE37745 (HR = 4.854, *p* = 0.030) (Figure 3G) was independent of age, sex, and disease stage. ROC curves for 1, 3, and 5 years were plotted to evaluate the predictive power of our signature. The areas under the curve (AUCs) for 1, 3, and 5 years were 0.681, 0.658, and 0.625, respectively, in the TCGA cohort (Figure 3E). The AUCs for 1, 3, and 5 years were 0.564, 0.607, and 0.598, respectively, in the GEO cohort (Figure 3H). The results of Kaplan–Meier curves and Cox regression analysis in the two subsets of the TCGA cohort (Figure 4) were consistent with these results in the entire discovery cohort and GSE37745. 95% Confidence interval (CI) of the AUCs in the TCGA and GEO cohorts are displayed in Supplementary Figure 1.

**TABLE 2:** Clinicopathological parameters of TCGA cohort and GEO cohort.

Characteristics	TCGA cohort (n = 479)	GEO cohort (n = 196)
Age (years), n (%) <65 ≥ 65	213 (44.47) 266 (55.53)	94 (47.96) 102 (52.04)
Gender, n(%)		
Male	219 (45.72)	107 (54.59)
Female	260 (54.28)	89 (45.41)
Stage, n (%)		
I	259 (54.07)	130 (66.33)
II	117 (24.43)	35 (17.86)
III	78 (16.28)	27 (13.78)
IV	25 (5.22)	4 (2.04)
Survival status, n (%)		
Dead	177 (36.95)	51 (26.02)
Alive	302 (63.05)	145 (73.98)

TCGA, the cancer genome atlas; GEO, gene expression omnibus.

### The Extracellular Matrix-Related Score Was Related to Clinical Variables

**FIGURE 5:**
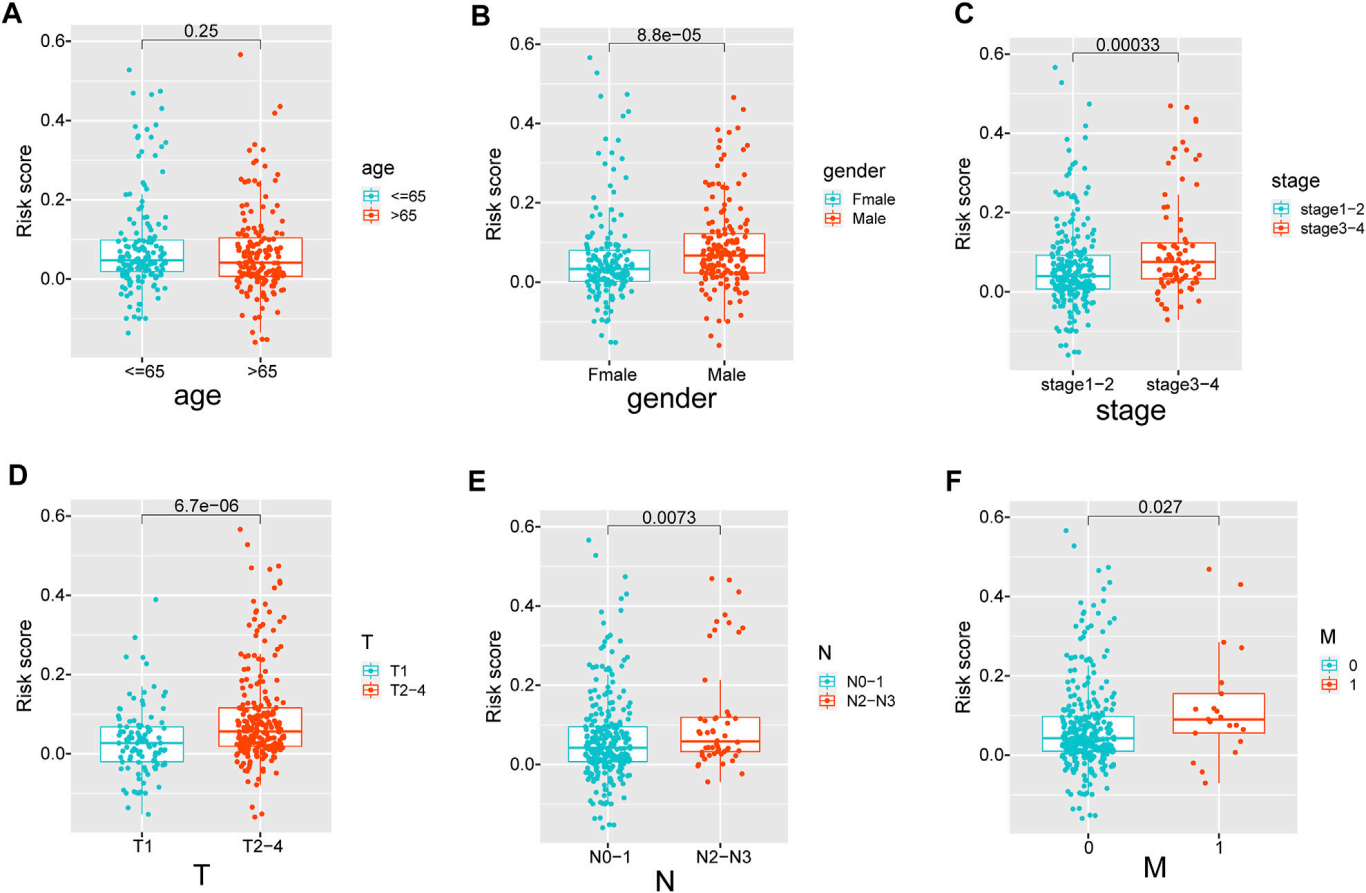
**(A**–**F)** The difference in ECMRS between patients stratified by clinicopathological variables. ECMRS = extracellular matrix-related score.

**FIGURE 6:**
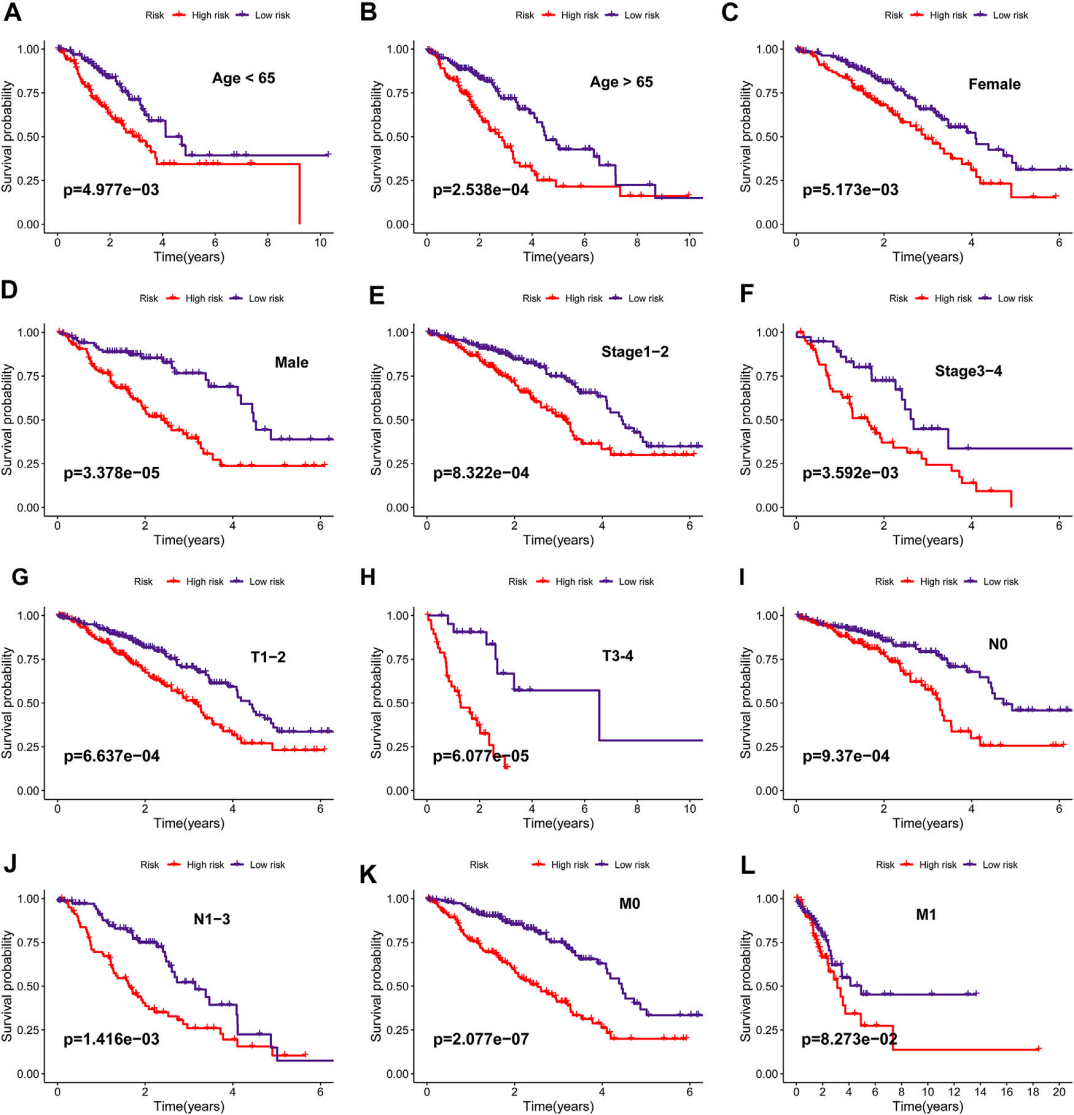
**(A**–**L)** Kaplan–Meier survival curves of overall survival between high- and low-risk groups in patients stratified by clinicopathological variables (age, sex, stage, T stage, N stage, M stage).

The difference in ECMRS in patients stratified by clinicopathological features was evaluated to show the connection between the prognostic signature and progression of LUAD (Figure 5). The results demonstrated that ECMRS was related to sex (*p* < 0.001), T stage (*p* < 0.001), N stage (*p* = 0.0073), and M stage (*p* = 0.027). Male patients and patients in the advanced tumor, node, and metastasis (TNM) stage had higher ECMRS. Survival curves of patients stratified by clinicopathologic features suggested that survival time was longer in patients with low ECMRS than in those with high ECMRS, and the prognostic value of the signature was not affected by clinical parameters (Figure 6).

### The Prognostic Signature can Affect Tumor Immunity

**FIGURE 7:**
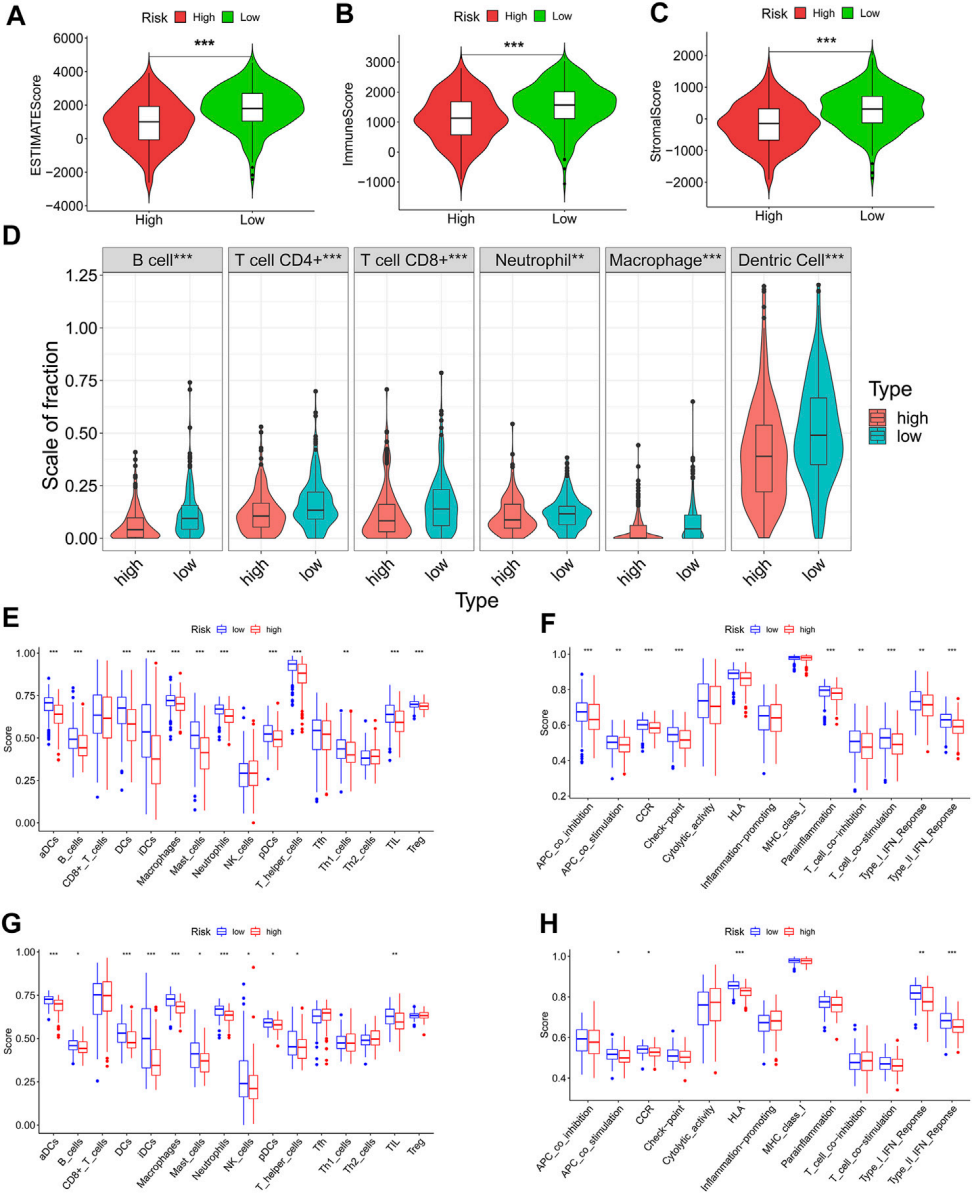
Differences in immune cells and immune functions between the high- and low-risk groups **(A-C)** ESTIMATE score, immune score, and stromal score between the high- and low-risk groups **(D)** Infiltration degree of immune cells between the high- and low-risk groups in TCGA cohort based on TIMER **(E)** Fraction of immune cells between the high- and low-risk groups based on ssGSEA in the TCGA cohort **(F)** Immune functions between the high- and low-risk groups based on ssGSEA in the TCGA cohort **(G)** Fraction of immune cells between the high- and low-risk groups based on ssGSEA in the GEO cohort **(H)** Immune functions between the high- and low-risk groups based on ssGSEA in the GEO cohort. TCGA = The Cancer Genome Atlas; GEO = Gene Expression Omnibus; *p < 0.05; **p < 0.01; ***p < 0.001.

The ECM is a vital determinant of antitumor immunity in solid tumors. Here, we speculated that our prognostic signature was associated with tumor immunity in patients with LUAD. To verify this speculation, we obtained the immune score, stromal score, and ESTIMATE score of each patient and found that the low-risk patients had significantly higher ESTIMATE score (*p* < 0.001) (Figure 7A), immune score (*p* < 0.001) (Figure 7B), and stromal score (*p* < 0.001) (Figure 7C) than the high-risk patients, which meant that the low-risk patients had higher degree of infiltration of antitumor immune cells and lower tumor purity in the TME than high-risk patients. Although the immune score was higher in patients with low ECMRS than in those with high ECMRS, the fraction of each immune component between the two groups remains unknown. Therefore, fractions of tumor-infiltrating immune cells in patients with LUAD were acquired from TIMER to evaluate their relationship with ECMRS. Patients with low ECMRS had higher degree of infiltration of CD8^+^ T cells (*p* < 0.001), CD4^+^ T cells (*p* < 0.001), B cells (*p* < 0.001), neutrophils (*p* < 0.001), macrophages (*p* < 0.001), and dendritic cells (DCs) (*p* < 0.001) than those with high ECMRS (Figure 7D). Next, to confirm the difference in immune cells present in the TME between the low- and high-risk patients and to identify the immune processes involved in the prognostic signature, ssGSEA was conducted in TCGA and GSE37745. The ssGSEA results suggested that patients with low ECMRS had more B cells, dendritic cells, macrophages, neutrophils, and mast cells in the TME than those with high ECMRS in both the TGGA (Figure 7E) and GEO cohorts (Figure 7G). The low-risk patients also had higher expression of chemokine receptors and human leukocyte antigen, and stronger response to antigen-presenting cell co-stimulation and interferon than the high-risk patients in both the TCGA cohort (Figure 7F) and GSE37745 (Figure 7H).

**FIGURE 8:**
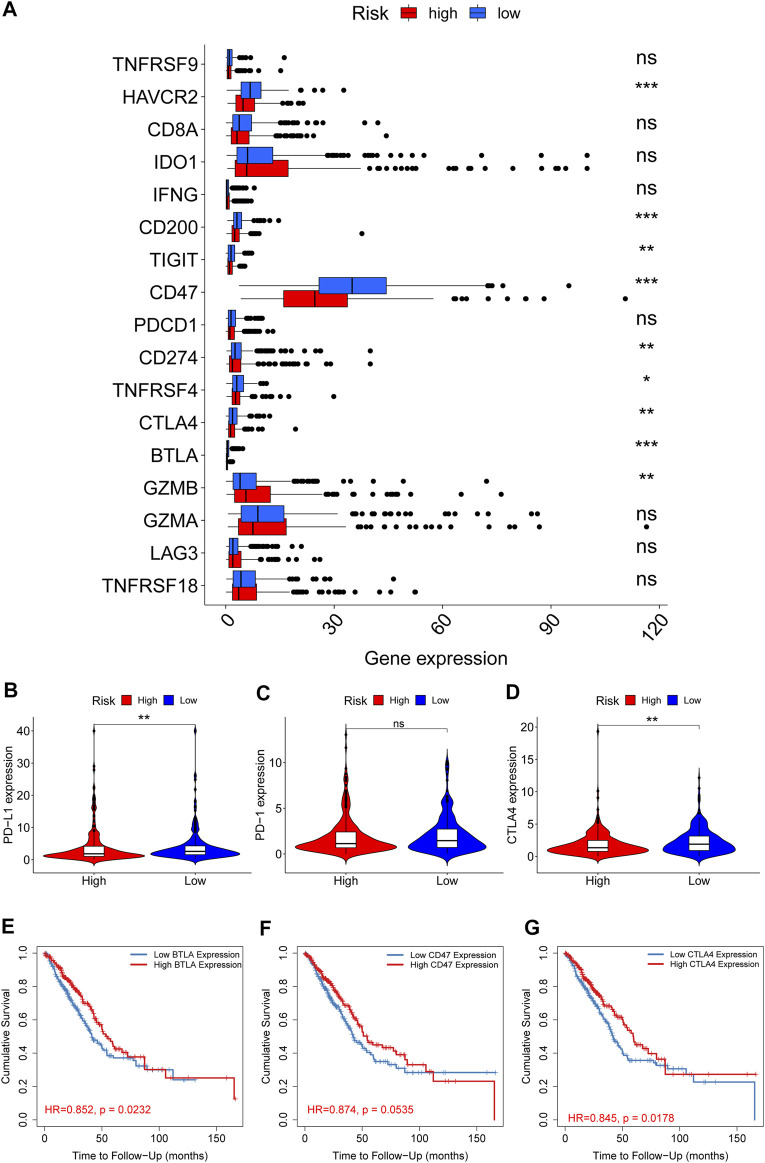
Differences in the expression of immune checkpoint genes between the high- and low-risk groups **(A)** Box plots show the difference in the expression of immune checkpoint genes between the high- and low-risk group **(B–D)** Violin plots show the difference in the expression of PD-L1, PD-1, and CTLA4 between the high- and low-risk groups **(E–G)** Survival curves of BTLA, CD47, and CTLA4 in TIMER. *p < 0.05; **p < 0.01; ***p < 0.001.

We also illustrated the relationship between ECMRS and the expression of key immune checkpoint genes. Patients with low ECMRS had higher expression of PD-L1, CTLA4, TIM3, and BTLA (Figures 8A, B, D) than those with low ECMRS, whereas no significant difference was observed in the expression of PD-1 between the two groups (Figure 8C). Among the immune checkpoint genes that were associated with ECMRS, the expressions of BTLA (HR = 0.852, *p* = 0.0232) (Figure 8E), CD47 (HR = 0.874, *p* = 0.0535) (Figure 8F), and CTLA4 (HR = 0.845, *p* = 0.0178) (Figure 8G) were associated with survival. High expression of CTLA4, BTLA, and CD47 was observed in low-risk patients and was positively related to survival duration, which confirmed our finding that ECMRS is an indicator of poor survival in patients with LUAD.

### Establishment of a Nomogram

**FIGURE 9:**
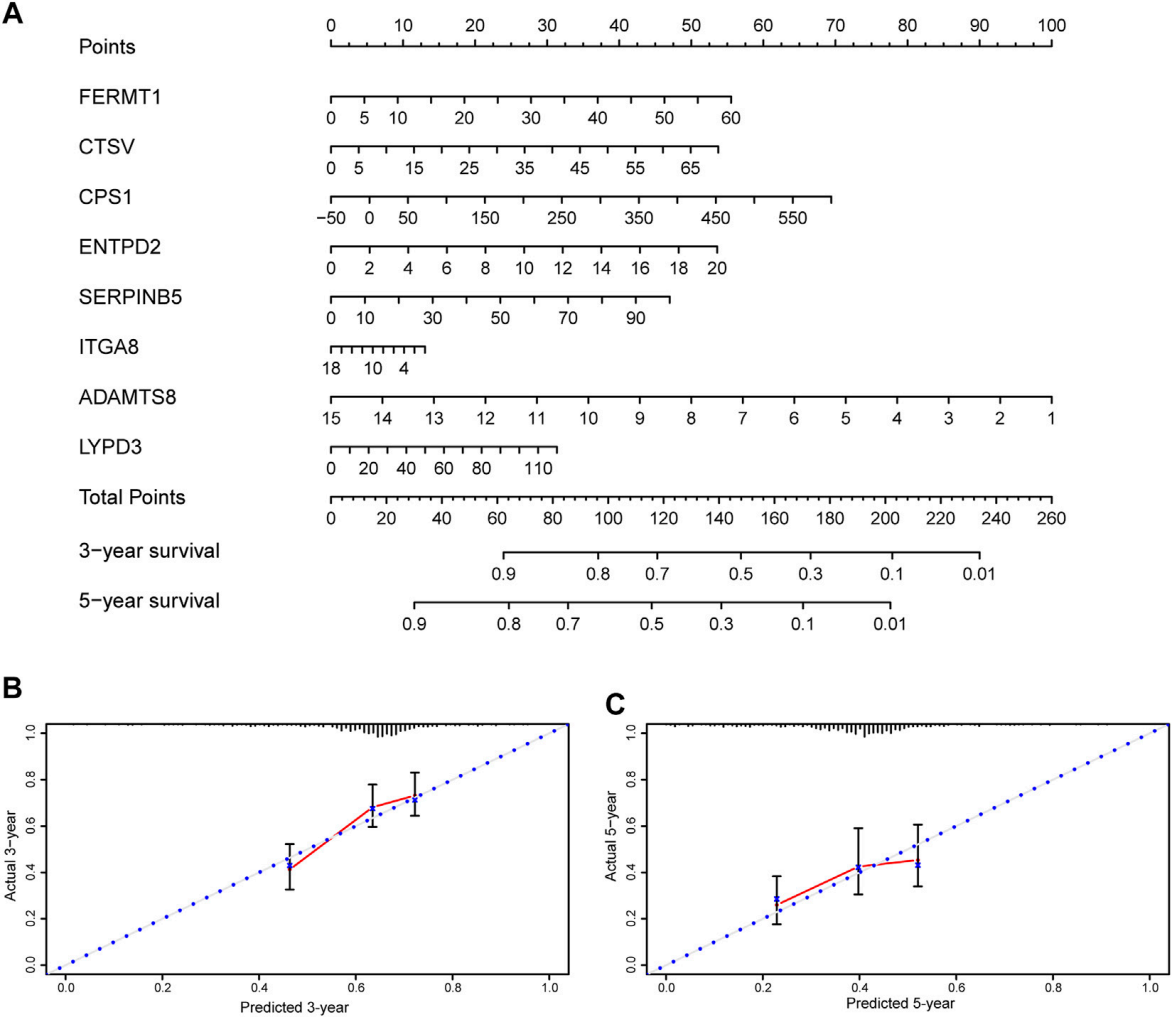
Nomogram and calibration plots of the prognostic signature **(A)** Nomogram based on eight prognostic ERGs predicting the overall survival probability of patients with lung adenocarcinoma **(B,C)** The three- and five-years calibration plots of the nomogram. ERGs = extracellular matrix-related genes.

A nomogram was constructed to visualize the prognostic signature, providing a reference for clinical applications (Figure 9A). Calibration curves at 3 and 5 years indicated that the nomogram could accurately predict OS (Figures 9B,C).

### Validation of the Prognostic Extracellular Matrix-Related Genes in Clinical Specimens and Multiple Databases

**FIGURE 10:**
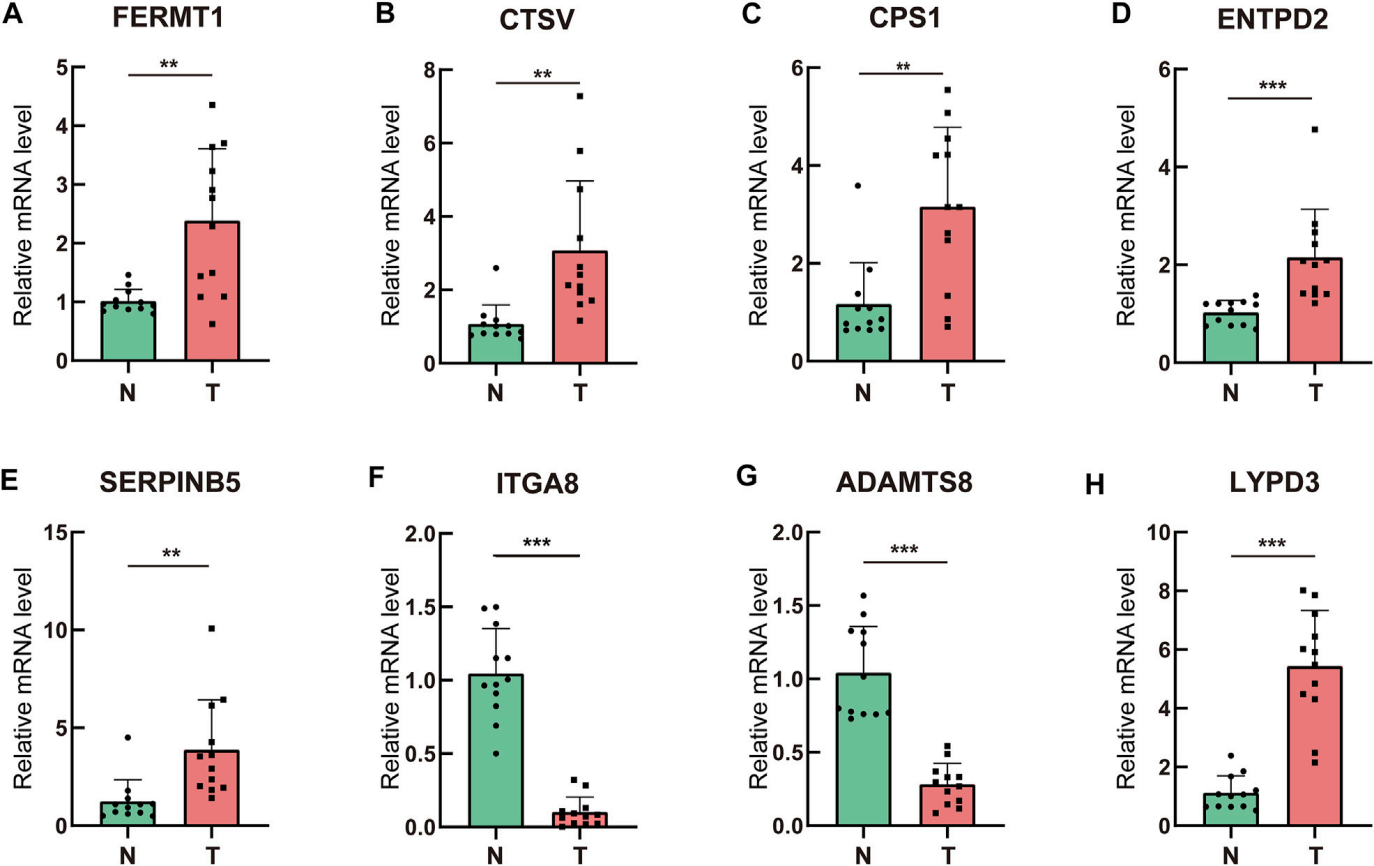
Validation of the prognostic ERGs in clinical specimens by qRT-PCR **(A–H)** Differential expression of the prognostic ERGs between lung adenocarcinoma tissues and paired adjacent normal lung tissues.ERGs = extracellular matrix-related genes; * *p* < 0.05; **p < 0.01; ***p < 0.001.

To assess the differential expression of the prognostic ERGs, polymerase chain reaction was conducted in 12 paired LUAD and peritumoral lung tissues collected at our institute. *ITGA8* and *ADAMTS8* were downregulated in tumor tissues, whereas *FERMT1*, *CTSV*, *CPS1*, *ENTPD2*, *SERPINB5*, and *LYPD3* were upregulated in tumor tissues (Figure 10).

Differential expression of the prognostic ERGs was also validated in the GSM43458 and GSM32863 datasets (Supplementary Figure 2), which is consistent with the polymerase chain reaction results. The immunohistochemistry images of *ENDPT2* , *FERMT1* , *SERPINB5*, *ITGA8*, *ADAMTS8* and *CPS1* were collected from HPA, which further verified differential expression of these prognostic genes between LUAD and normal lung tissues **(** Supplementary Figure 3 **)**. However, ADAMTS8 were not detected in both LUAD and normal lung tissues, which may be attributed to its low expression. The prognostic value of the eight genes was validated using the Kaplan–Meier plotter. Patients with high expression of *ITGA8* or *ADAMTS8* survived longer than those with low expression of *ITGA8* or *ADAMTS8*, whereas high expression of *FERMT1*, *CTSV*, *CPS1*, *ENTPD2*, *SERPINB5*, and *LYPD3* was associated with shorter survival duration (Supplementary Figure 4).

## Discussion

The ECM is a fundamental component of the TME, and its alterations can affect the phenotypes and immune environment of cancer cells. Perturbation of the biochemical and mechanical properties of the ECM can affect cell behavior through transmembrane receptors, such as integrins and syndecans. Desmoplastic response is common in solid tumors and is characterized by excessive deposition of ECM proteins, which has been suggested to be a feature of poor prognosis (Sundqvist et al., 2020). Cancer cells have to migrate through the ECM to spread to other parts of the body; therefore, metastasis can be affected if the biophysical properties of the ECM, such as deformability or stiffness, are changed. Remodeled and stiffened ECM has been shown to promote the dissemination of cancer cells (Han et al., 2016; Miroshnikova et al., 2017). Furthermore, the ECM is a reservoir for cytokines and controls their distribution and interaction with cells (Huleihel et al., 2016). In addition, ECM can serve as a protective shield against host antitumor immunity in solid tumors, thereby impeding the infiltration of lymphocytes and reducing the efficacy of immunotherapy. Changes in TME and tumor behavior can be fully reflected by perturbations in the ECM. Thus, ECM-related biomarkers have enormous potential for prognostication.

In this study, eight key prognostic ERGs (*FERMT1*, *CTSV*, *CPS1*, *ENTPD2*, *SERPINB5*, *ITGA8*, *ADAMTS8*, and *LYPD3*) were identified by stepwise statistical analyses to construct a prognostic signature for patients with LUAD. Among these genes, *ITGA8* and *ADAMTS8* were downregulated in LUAD tissues and functioned as tumor suppressor genes, whereas the remaining genes were oncogenes. Although only few studies have highlighted the functions of these eight genes in the development of LUAD, their vital function in other cancers has been delineated by numerous studies. *FERMT1* encodes the kindlin-1 protein, which mediates integrin activation and cell adhesion. Evidence has shown that kindlin-1 facilitates integrin-mediated TGF-β activation (Rognoni et al., 2014). Upregulation of *FERMT1* promotes the progression of gastric cancer (Fan et al., 2020). Liu et al. found that *FERMT1* was overexpressed in colon adenocarcinoma, promoting epithelial-mesenchymal transition and metastasis (Liu et al., 2017). Yan et al. found that *FERMT1* is overexpressed in esophageal cancer and facilitates the proliferation of cancer cells (Yan et al., 2019). Cathepsin V (*CTSV*/*CTSL2*) is a cysteine proteinase that can degrade some constituents of the ECM and has been found to be related to the malignancy of tumor cells and the prognosis of patients with breast cancer (Toss et al., 2020; Wang et al., 2020). Carbamoyl-phosphate synthetase 1 (*CPS1*) not only serves as a crucial catalyst in the urea cycle but also functions in the progression of cancer. Studies have demonstrated that *CPS1* is downregulated in hepatocellular carcinoma and that its decline could lead to poor survival (Ridder et al., 2021). *CPS1* has also been identified as a biomarker of progression in colorectal cancer (Palaniappan et al., 2016). *ENTPD2* was found to be elevated in hepatocellular carcinoma, indicating an unfavorable prognosis for patients. Additionally, *ENTPD2* has been proven to impede the differentiation of myeloid-derived suppressor cells, which can induce immunosuppression in hepatocellular carcinoma. Inhibition of *ENTPD2* could augment the efficacy of immunotherapy (Chiu et al., 2017). A disintegrin and metalloproteinase with thrombospondin motifs 8 (*ADAMTS8*) is a secreted protein that functions in the degradation of the ECM. Similar to matrix metalloproteinases, adamalysins are enzymes that can cause degradation and crosslinking of the ECM. *SERPINB5* belongs to the serpin superfamily which can regulate degradation of structural elements of the ECM such as collagens and hyaluronan. *SERPINB5* has been reported to mediate invasion of cancer cell and identified as an oncogene in multiple tumors (Chang et al., 2018; Atay, 2020). *ADAMTS8* has been reported to function as a tumor suppressor gene in various solid tumors (Zhao et al., 2018; Li et al., 2020b; Wu et al., 2020). In our study, *ADAMTS8* was downregulated in LUAD tissues and correlated with favorable outcomes, which is consistent with previous findings. ITGA8 belongs to the ITGA subfamily of integrins. Apart from mediating cell-cell and cell-ECM adhesion, integrins also play a pivotal role in signal transduction and modulate various cellular processes (Zhang and Wang, 2012). *ITGA8* has been proven to be correlated with favorable outcomes in patients with basal-like and HER2+ breast cancer and colon cancer (Gong et al., 2019; Rojas et al., 2021).

The effect of the prognostic signature developed in this study on survival was independent of age and disease stage. Although age, disease stage, and ECMRS are all contributing factors to OS, the HR of ECMRS in univariate and multivariate Cox regression analysis was far greater than that of age and disease stage, which indicated the superiority of ECMRS as a stratification tool for survival in LUAD. Till date, the TNM staging system is the major tool used for prediction of the survival of patients with LUAD. However, with in-depth understanding of tumor behavior and updating of antitumor treatment, the TNM staging system is unable to meet clinical demands. The prognostic signature developed in this study may therefore be applicable as a supplement to the TNM staging system.

Immune checkpoint inhibitors (ICIs) are effective antitumor therapies. However, only 40–45% of non-small-cell lung carcinoma patients achieve remission after administration of ICIs (Borghaei et al., 2015; Garon et al., 2015; Larkin et al., 2015), indicating that most patients cannot benefit from ICIs. In solid tumors, dense ECM can serve as a protective shield against host antitumor immunity. Excessive matrix crosslinking can prevent immune cells and immunotherapeutic drugs from reaching the TME (Henke et al., 2019). Therefore, the expression of ERGs may affect tumor immunity to some extent. Indeed, the prognostic signature developed in our study was shown to predict tumor immunity in LUAD. Patients with low ECMRS had higher degree of immune cell infiltration, including CD8^+^ T cells, CD4^+^ T cells, and macrophages. Thus, our prognostic signature can serve as a useful tool to predict tumor immunity, and targeting the genes in the signature may collaborate with ICIs to exert antitumor efficacy.

Our research has a few limitations. First, the prognostic signature was retrospectively constructed and validated in TCGA and GEO databases; therefore, a prospective cohort study is needed to verify our findings. Second, the mechanism underlying the signature has not been explored, and *in vivo* and *in vitro* experiments should be conducted to elucidate the mechanism of the prognostic ERGs in oncogenesis and tumor immunity.

In conclusion, we constructed a gene signature and developed a scoring system based on the expression of prognostic ERGs which can predict the survival and tumor immunity of patients with LUAD. Our study contributes to dissection of the ECM in LUAD and identifies promising prognostic indicators and potential therapeutic targets for patients with LUAD.

## Data Availability

The datasets presented in this study can be found in online repositories. The names of the repository/repositories and accession number(s) can be found in the article/Supplementary Material.

## Abbreviations

ECM, extracellular matrix; LUAD, lung adenocarcinoma; TME, tumor microenvironment; ERG, extracellular matrix-related gene; TCGA, The Cancer Genome Atlas; LASSO, Least Absolute Shrinkage and Selection Operator; GEO, Gene Expression Omnibus; CI, confidence interval; HPA, Human Protein Atlas; ECMRS, extracellular matrix-related score; FDR, false discovery rate; OS, overall survival; DC, dendritic cell; HR, hazard ratio; CI, confidence interval.
