# Supplementary figures and images for: Integrative Analysis Constructs an Extracellular Matrix-Associated Gene Signature for the Prediction of Survival and Tumor Immunity in Lung Adenocarcinoma
Source: Front Cell Dev Biol. 2022 Apr 26;10:835043. doi: 10.3389/fcell.2022.835043 (PMC9086365; doi:10.3389/fcell.2022.835043)

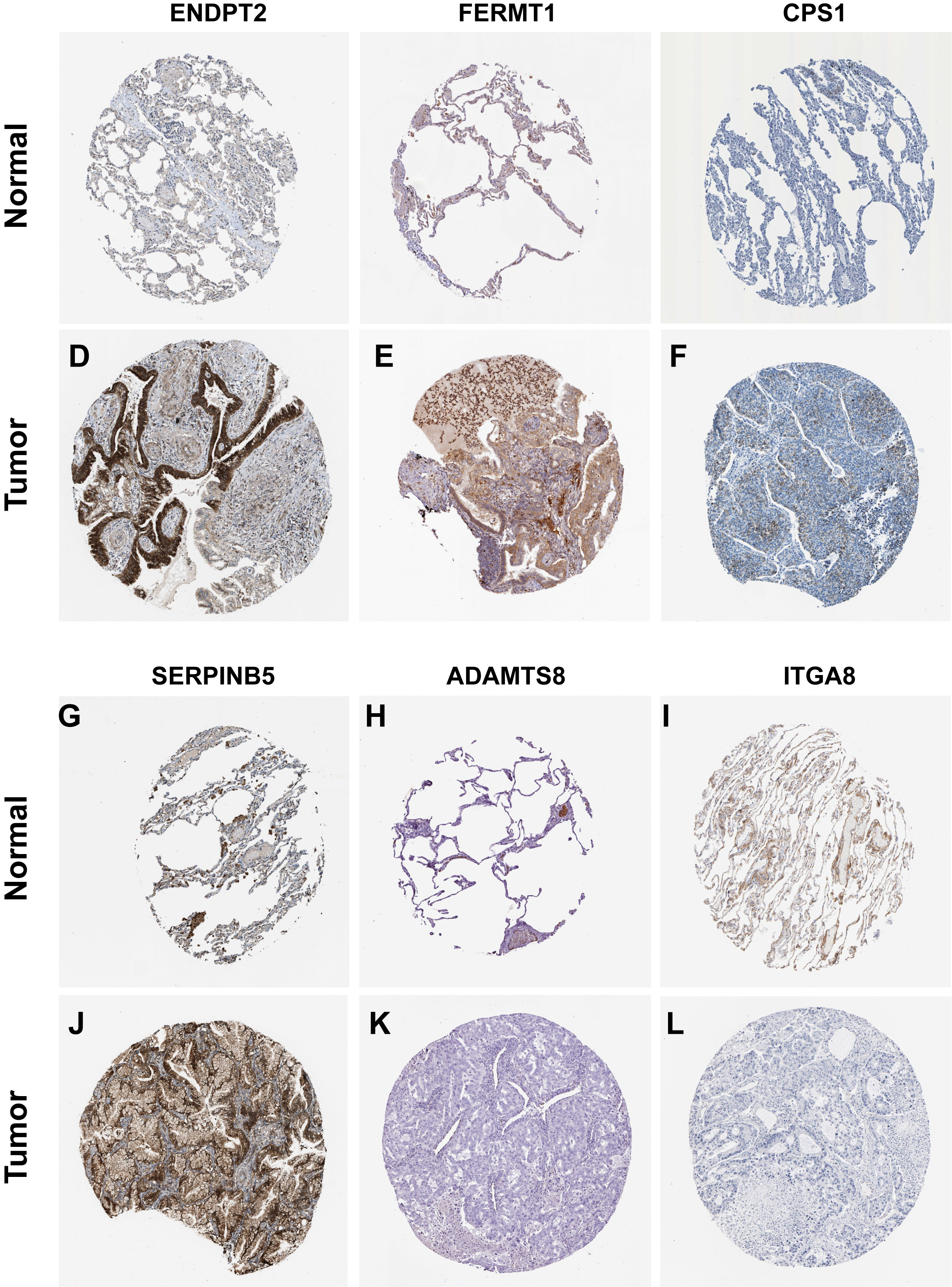

Supplement: Supplementary file 1 [file Image3.TIF]

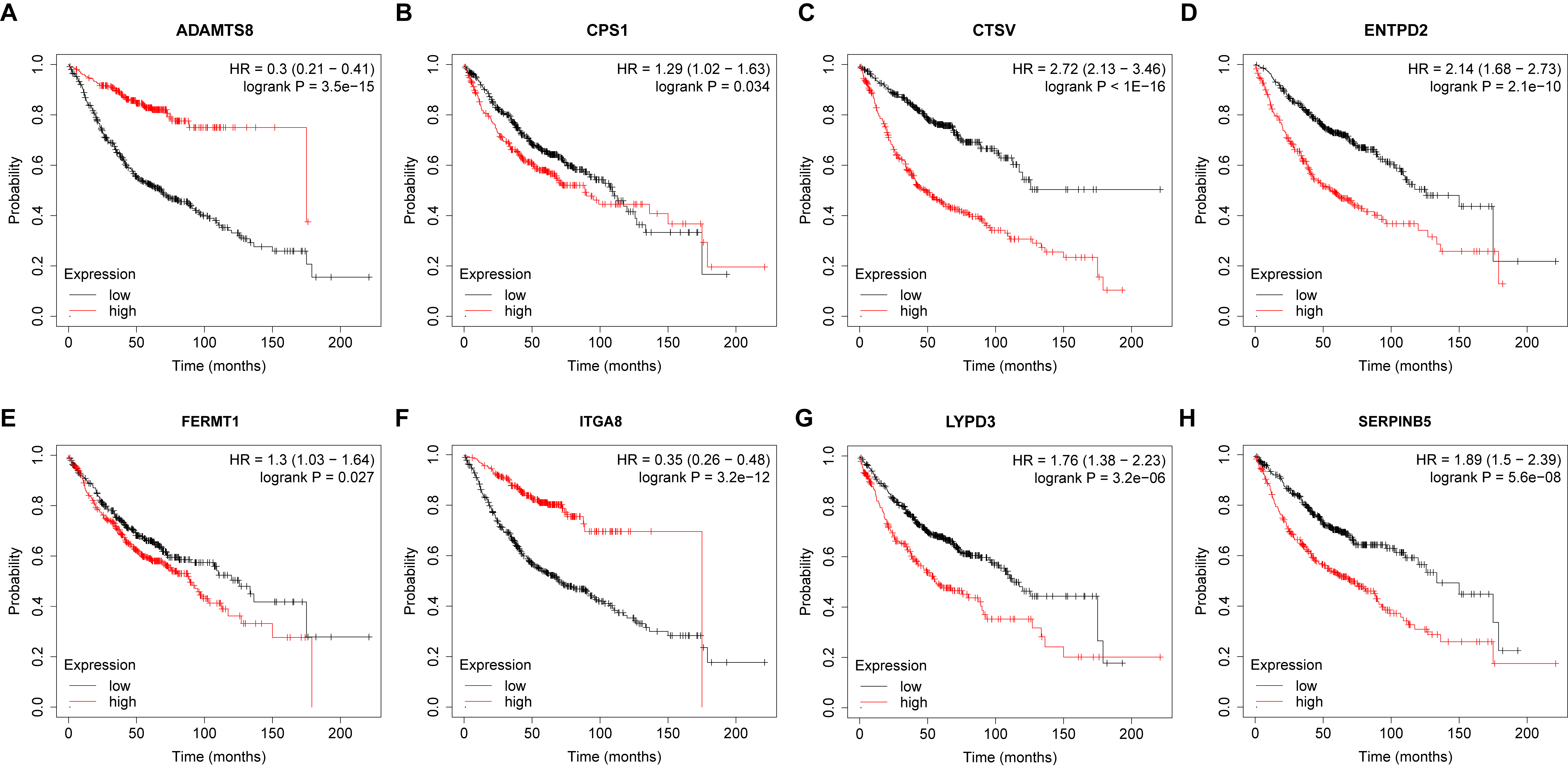

Supplement: Supplementary file 2 [file Image4.TIF]

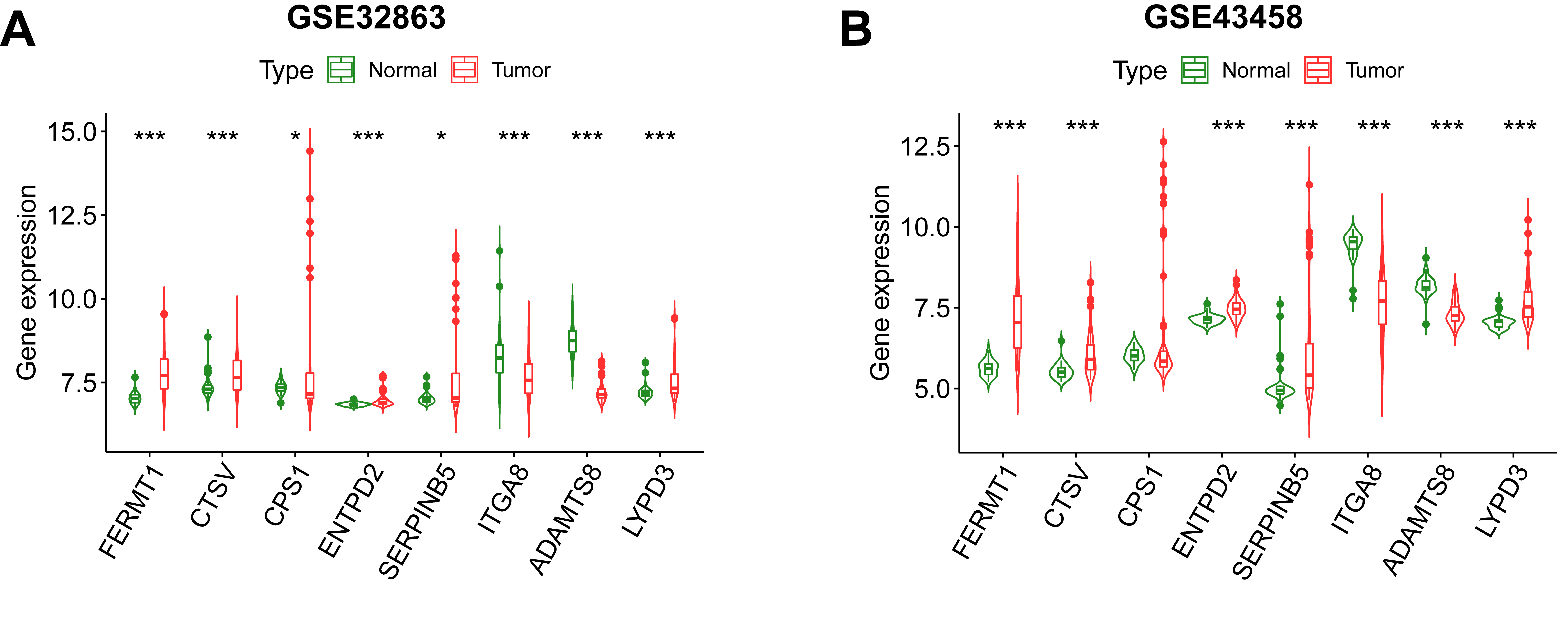

Supplement: Supplementary file 3 [file Image2.TIF]

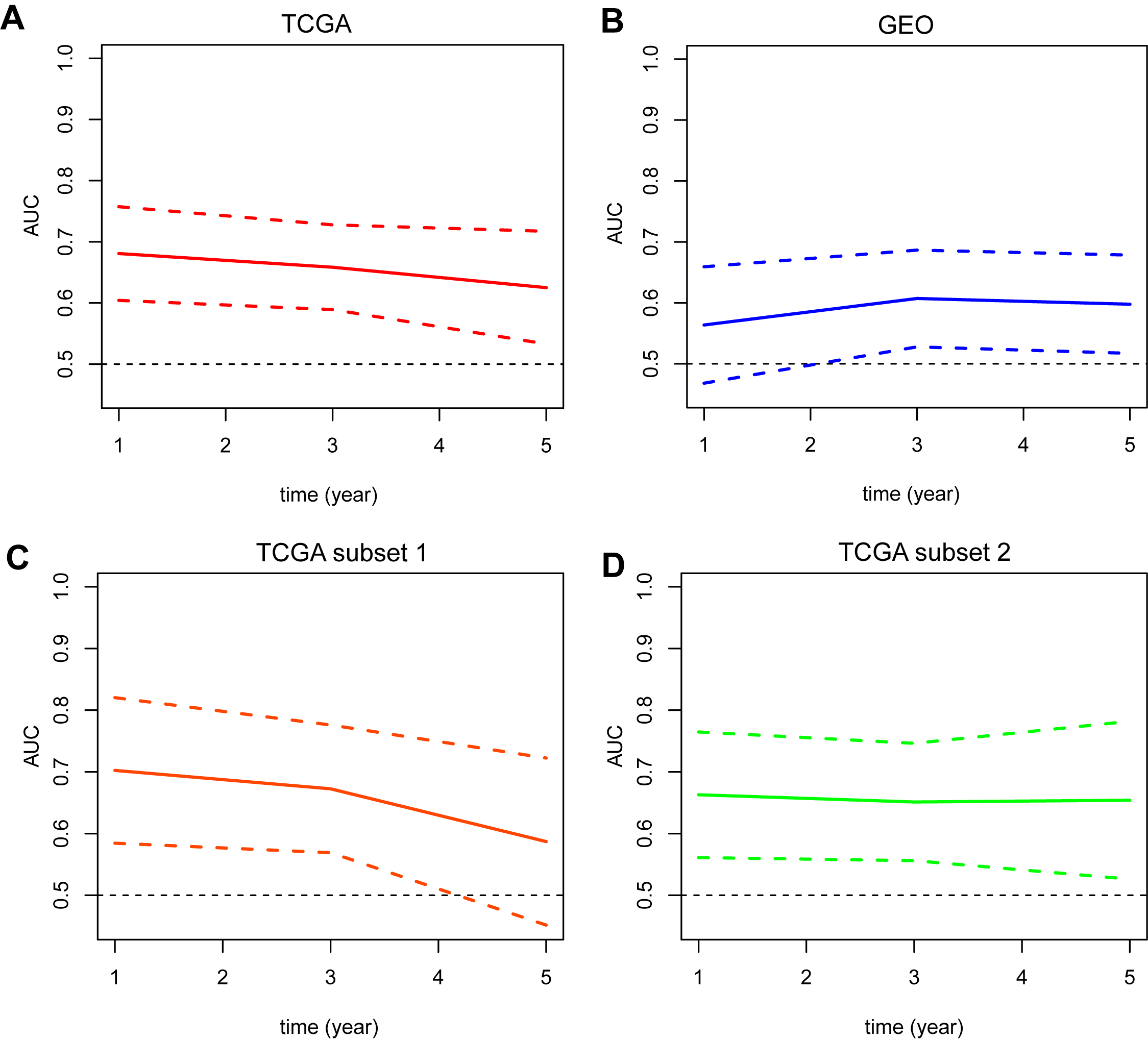

Supplement: Supplementary file 4 [file Image1.TIF]
