# Supplementary material for: Integrative Analysis Constructs an Extracellular Matrix-Associated Gene Signature for the Prediction of Survival and Tumor Immunity in Lung Adenocarcinoma
Source: Front Cell Dev Biol. 2022 Apr 26;10:835043. doi: 10.3389/fcell.2022.835043 (PMC9086365; doi:10.3389/fcell.2022.835043)
Supplement: Supplementary file 5 [file Table2.DOCX]

Supplementary Table 2:Primer sequence of the prognostic ERGs.

| Gene | Forward-primer | Reverse-primer |
| --- | --- | --- |
| FERMT1 | TTGAAGATGGTGAGGTTGCGAGTC | GGGTTGGCTGAATGCGAGGATG |
| CTSV | GGTGTTCTGGTGGTTGGCTACG | CACAGTGGTTGTTCTTGTCTTTGGC |
| CPS1 | CCCCGTCAATTAGACTATGACA | CATTGTTGTCTGTGTCGATCTG |
| ENTPD2 | CCTGGACGCTGGTTCTTCACAC | GGGTTGTCTGCATAGCTGGAGATG |
| SERPINB5 | ATGCCAAGGTCAAACTCTCCATTCC | CAGCCCTAGATTTTCCAGACAAGCC |
| ITGA8 | AGATCCTCACTGGCACCGAGAC | AAGGCACTCCGATGGCAATGTC |
| ADAMTS8 | ATGTTCTCGGACCTGTGGAGGAG | CGTGTGGCATGACTGGTACTTGG |
| LYPD3 | CCTCAACCACATCTGTCACCACTTC | AGTCAACCTGGGCTCCTCATCC |
