# Supplementary material for: Integrative Analysis Constructs an Extracellular Matrix-Associated Gene Signature for the Prediction of Survival and Tumor Immunity in Lung Adenocarcinoma
Source: Front Cell Dev Biol. 2022 Apr 26;10:835043. doi: 10.3389/fcell.2022.835043 (PMC9086365; doi:10.3389/fcell.2022.835043)
Supplement: Supplementary file 7 [file Table3.DOCX]

Supplementary Table 3: Genes and coefficients of the prognostic signature.

| Gene | Coef |
| --- | --- |
| FERMT1 | 0.007877 |
| CTSV | 0.001558 |
| CPS1 | 0.000367 |
| ENTPD2 | 0.014475 |
| SERPINB5 | 0.00094 |
| ITGA8 | -0.01916 |
| ADAMTS8 | -0.00508 |
| LYPD3 | 0.003902 |
